# A comparison of ultrasound guided bilateral single injection shot Erector Spinae Plane blocks versus wound infiltration for post-operative analgesia in laparoscopic assisted colonic surgery- a prospective randomised study

**DOI:** 10.1186/s12871-021-01474-8

**Published:** 2021-10-26

**Authors:** V. Rao Kadam, G. Ludbrook, R. M. van Wijk, P. Hewett, V. Thiruvenkatarajan, S. Edwards, P. Williams, S. Adhikary

**Affiliations:** 1 Acute Care Medicine, The University of Adelaide, The Queen Elizabeth Hospital, Adelaide, SA Australia; 2 Department of Anaesthesia, The Queen Elizabeth Hospital, 28 Woodville Road, Woodville South, SA 5011 Australia; 3 Acute Care Medicine, University of Adelaide, Royal Adelaide Hospital, Adelaide, SA Australia; 4 Department of Surgery, University of Adelaide, The Queen Elizabeth Hospital, Adelaide, SA Australia; 5 Adelaide Health Technology Assessment, School of Public Health, University of Adelaide, Adelaide, SA Australia; 6 Department of Anesthesiology and Perioperative Medicine, Penn State Hershey Medical Center, Penn State College of Medicine, Hershey, PA USA

**Keywords:** Ultrasound, Erector Spinae Plane, Post-operative analgesia, Local anaesthetic

## Abstract

**Background:**

Both wound infiltration (WI) with local anaesthetic and Erector Spinae Plane block (ESPB) have been described for post-operative analgesia after abdominal surgery. This study compared the efficacy of WI versus ESPB for post-operative analgesia after laparoscopic assisted colonic surgery.

**Methods:**

Seventy-two patients between 18 and 85 years of age undergoing elective surgery were randomised to receive either WI or ESPB. In the WI group a 40 ml bolus of 0.5% Ropivacaine, infiltrated at the ports and minimally invasive wound at subcutaneous and fascia layers. In the ESPB group at T8 level, under ultrasound guidance, a 22-gauge nerve block needle was passed through the Erector Spinae muscle to reach its fascia. A dose up to 40 ml of 0.5% Ropivacaine, divided into two equal volumes, was injected at each side. Both groups had a multimodal analgesic regime, including regular Paracetamol, dexamethasone and patient-controlled analgesia (PCA) with Fentanyl. The primary end point was a post-operative pain score utilising a verbal Numerical Rating Score (NRS, 0–10) on rest and coughing in the post anaesthetic care unit (PACU) and in the first 24 h. Secondary outcomes measured were: opioid usage, length of stay and any clinical adverse events.

**Results:**

There was no significant treatment difference in PACU NRS at rest and coughing (*p*-values 0. 382 and 0.595respectively). Similarly, there were no significant differences in first 24 h NRS at rest and coughing (*p*-values 0.285 and 0.431 respectively). There was no significant difference in Fentanyl use in PACU or in the first 24 h (*p*- values 0.900 and 0.783 respectively). Neither was there a significant difference found in mean total Fentanyl use between ESPB and WI groups (*p*-value 0.787).

**Conclusion:**

Our observations found both interventions had an overall similar efficacy.

**Trial registration:**

The study was registered with the Australian New Zealand Clinical Trial Registry (ACTRN: 12619000113156).

## Background

The Erector Spinae Plane block (ESPB) was first described by Forero, in 2016 [1]. Initially, the block was performed for thoracic and breast surgery and its use has now been reported for abdominal surgery [2–4]. This block has gained popularity in the last 5 years, as one of the options for post-operative pain relief after abdominal surgery [2–4]. Both single bolus injection and catheter technique have proven to be beneficial as part of multimodal analgesia in surgeries involving the thorax and abdomen [5–8]. The technique involves injecting local anaesthetic (LA) into the myofascial plane beneath the fascia covering the Erector Spinae muscle using real time ultrasound guidance. This approach is gaining popularity mainly due to its simplicity in performance. It is relatively easy to visualise the para spinal muscles at the mid thoracic about 3 cm lateral to the midline. Clinical trials reported to be effective in use of ESPB in laparoscopic cholecystectomy [9–11] but not in laparoscopic colonic surgery.

The purpose of this study was to assess the efficacy of single injection ESPB performed for post-operative analgesia in laparoscopic assisted colonic surgery. Efficiency was assessed by comparing pain scores. We hypothesized that ultrasound guided ESPB is superior to wound infiltration performed at the end of surgery in providing pain relief without major side effects.

## Methods

The study was conducted at The Queen Elizabeth Hospital (TQEH), part of Central Adelaide Local Health Network (CALHN) between January 2019 and September 2020. The study was registered with the Australian and New Zealand Clinical Trials Registry (ACTRN12619000113156 date 24/01/2019). Institutional Human Ethics and Research Committee (HREC/18/CALHN/456) approval was obtained and all patients provided prior informed consent for their participation in the study.

The primary end point was post-operative pain score utilising a verbal Numerical Rating Score (NRS, 0–10) on rest and coughing in PACU and during the first 24 h (worst NRS on rest and coughing). Secondary outcomes measured were opioid usage until 24 h post-operatively, length of stay (days) and clinical determinants of adverse effects.

Patients with an American Society of Anesthesiologists Physical Status 1–3, greater than 18 years of age, and undergoing elective laparoscopic colonic surgery, were recruited for the study to receive either ESPB or WI at the end of surgery and before extubation. Patients were excluded if they had communication barriers, sensitivity or allergy to local anaesthetics, were pregnant, had a pre-operative daily use of opioids equivalent to 10 mg/day of morphine or above or if the procedure could not be performed laparoscopically. The study was designed with the groups randomised to the intervention allocation based on a computer-generated sequence.

All patients received a standardized general anaesthetic technique and monitoring. They were administered intermittent intravenous fentanyl as intra-operative opioid analgesia. At the end of procedure, before extubation, an ESPB was performed by an experienced anaesthetist or the WI was performed by the surgical fellow/consultant.

An in-plane approach in the lateral position was used under ultrasound guidance for the ESPB. T8 level was confirmed by counting the spinous process from T1 down to T8. Using a 6- to 15-MHz high-frequency linear probe (Sonosite X-Porte, SonoSite Inc. Bothell, WA, USA), the 2 muscle layers of the posterior spine anatomy, namely trapezius and erector spinae (ES) muscles, were visualized slightly cephalad to the T8 transverse process. The 22-gauge stimuplex (Pajunk, Geisingen, Germany) nerve block needle tip was placed deep to the ES muscle, beneath the fascia in a cephalad to caudal direction. Needle position was confirmed by a 3 ml normal saline test dose under ultrasound guidance to observe linear spread lifting the ES muscle. Ropivacaine (AstraZeneca Pty Ltd., Sydney, NSW, Australia) dissemination was confirmed lifting the ES muscle in real time under ultrasound guidance from start to completion of injection. A dose of 40 ml of 0.5% Ropivacaine (200 mg), divided into two equal volumes, was injected at each side. In the WI group 40 ml of 0.5% Ropivacaine was injected at the surgical ports and into the minimally invasive wound. In the PACU and subsequently in the wards for 24 h (time from PACU), patients were observed and questioned for signs and symptoms of local anaesthetic systemic toxicity (LAST), such as perioral numbness, tingling sensation, tinnitus, metallic taste, muscle twitching, and convulsions. Sensory block was assessed by recovery staff after surgery in PACU using a cold test on either side of the anterior abdomen between xiphi-sternum and pubic symphysis (dermatomes T6-L1).

All patients had a pre-operative ECG and a repeat ECG was to be performed if any signs and symptoms of LA toxicity were observed. Patients were administered Paracetamol 1 g QID (orally or IV) and received a single dose of Dexamethasone 8 mg intra-operatively as part of a multimodal analgesic approach. A Fentanyl PCA device (bolus 10 to 40 mcg based on age; lockout time 5 min; no background infusion) was provided as rescue analgesia. The difference in PCA usage was used as an indication of efficacy of the analgesic techniques. The primary endpoints measured were NRS for Pain at rest and on coughing in PACU at 0 and I hour and in the post-operative ward at 24 h. Other end points were Fentanyl use in PACU and first 24 h, any rescue medication used, procedure related technical issues, potential side effects or complications in relation to the technique used and length of stay (days). Data was entered in excel by the research assistant at the trial centre, who has blinded the statistician for group allocation.

### Statistical analysis

Continuous measures are presented as means with standard deviations and medians with interquartile ranges, based on the normality of their distribution. Categorical measures are presented as frequencies and percentages. Group comparisons on baseline characteristics were assessed using Student’s T-test, a Wilcoxon Rank Sum test, Pearson’s Chi-square statistic or Fisher’s Exact Test as required, and linear mixed-effects models were used to compare pain and fentanyl use between ESP and WI groups, across time periods, adjusting for repeated measurements over time. Linear regressions were also used for two fentanyl outcomes. All tests are two-tailed and assessed at the 5% alpha-level. The statistical software used was SAS 9.4 (SAS Institute Inc., Cary, NC, USA).

### Sample size

As RCTs for the use of ESP blocks in laparoscopic colonic surgery have not been published, an approximate scenario was established to obtain the required patient numbers. Calculations were based on the primary outcome (pain scores) and it was determined that a clinically meaningful difference between groups would be 2.5 points on the NRS. Assuming constant variance and a standard deviation of 3 points, a sample of 24 patients per group was required. The sample was inflated to 36 patients per group to account for intra-patient correlations arising from repeated measures. Thus, a total of 72 patients were required.

### Randomisation

The randomisation schedule was generated by the Clinical Trials Division of the Pharmacy Department at The Queen Elizabeth Hospital. To ensure equal distribution of the intervention arm, randomisation was done in specific blocks to pre-determined numbers known only to the clinical trials division. A simple randomisation table was created by computer software (computerised sequence generation). This allocation was concealed by a sealed opaque envelope. The proceduralist was unable to be blinded; however, the patients were blinded to group allocation. The person analysing the data was also blinded.

## Results

**Fig. 1:**
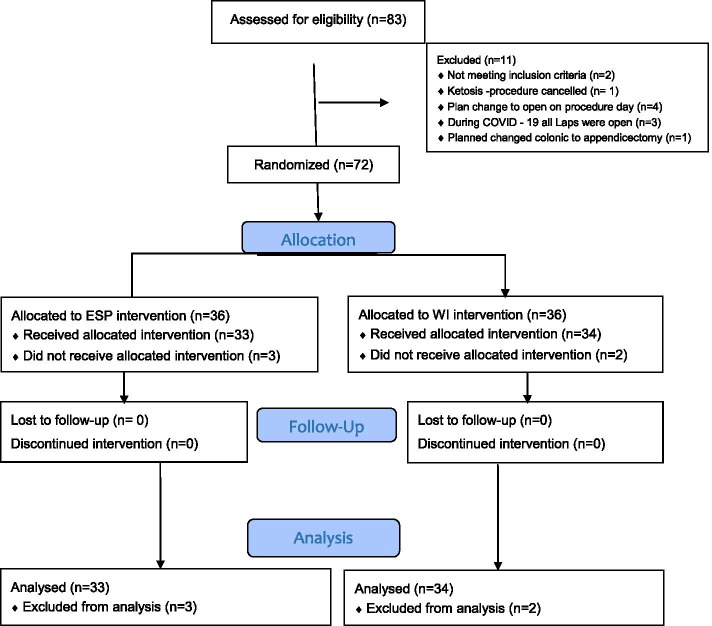
CONSORT Flow Diagram

Seventy-two patients were recruited. Five patients did not complete the study and 67 were included in the analysis. These five patients excluded from analysis had a breach of protocol and none were lost to follow up (see Consort flow diagram Fig. 1). Table 1 shows the patient demographics in each group. Wound infiltration time was significantly lower than ESPB (*p* = < 0.01), otherwise both the groups were comparable with respect to pre-operative status and operative specifications are shown in Table 1. No block related complications, such as vascular/visceral puncture or local anaesthetic toxicity were recorded. None of the patients had well defined dermatomal spread in the ESPB group in PACU. Only one patient had patchy spread. Table 2 shows the pain scores and fentanyl use with mean and standard deviation by technique and time period, mean differences, 95% confidence intervals (CI) and comparison and global *P* values. There were no significant differences between the groups on intra-operative fentanyl use or total fentanyl use. There were also no significant differences between the groups for rest or cough pain scores or cumulative fentanyl use in PACU or on day one (refer to Table 2). The mean differences between ESP and WI groups for rest and cough pain ranged from − 0.6 to − 0.3 were not significant. Table 3 shows the complications. There was no difference in the complication incidences between the groups. Technically, we did not have any failures but had slight difficulty in three obese participants in the ESPB group requiring 120 mm needles to reach the plane. None of the patients had any sign or symptoms of LAST in the 24- h study period. However, 3 patients developed tachycardia after 48 h which was related to low haemoglobin requiring transfusion and anastomotic leak requiring intervention. One patient developed bradycardia (50/min) in the ESP group at 24 h on the ward, but remained stable. The average theatre time for (LA loading and checking/positioning/setup ultrasound equipment) was 20 min for ESP group compared to 10 min in WI group.

**Table 1:** Patient demographics and details by technique

	**ESPB group**	**WI group**	*P*-value*
	***N* = 33**	***N* = 34**	
Age (years) mean (SD)	60.5 (17.8)	61.2 (13.3)	0.86
Gender			
Female	14 (52%)	13 (48%)	0.73
Male	19 (48%)	21 (52%)	
Weight (kg) mean (SD)	84.3 (14.6)	77.2 (17.5)	0.078
BMI (kg/ht.^2)	29.4 (5.4)	26.8 (5.7)	0.059
ASA status			
1	2 (50%)	2 (50%)	0.85
2	15 (54%)	13 (46%)	
3	16 (46%)	19 (54%)	
Operations			0.95
Hemicolectomy	14 (47%)	16 (53%)	0.70
Anterior resection	10 (50%)	10 (50%)	0.94
Hartmann’s	2 (100%)	0 (0%)	0.15
Reversal of Hartmann’s	1 (33%)	2 (67%)	0.57
Ultra low anterior resection	2 (50%)	2 (50%)	0.97
Ileocecal resection	1 (100%)	0 (0%)	0.31
Small bowel resection	0 (0%)	2 (100%)	0.16
Others	3 (60%)	2 (40%)	0.62
PACU time (mins) median (IQR)	60 (60, 105)	62 (45, 90)	0.61
Flatus time (mins) median (IQR)	48 (48, 72)	48 (48,72)	0.43
Bowel motion time (mins) median (IQR)	77 (72, 96)	72 (60, 120)	0.84
Hospital LOS (days) median (IQR)	5 (4, 7)	4 (4, 8)	0.92

Data are presented as mean (SD) or median (IQR) for continuous measures, and n (%) for categorical measures

*ESPB* denotes Erector Spinae Plane Block, *WI* denotes wound infiltration, *PACU* Post anaesthetic care unit, *mins* Minutes, *LOS* Length of stay

*Independent t-test *P* value, Wilcoxon Rank Sum Test *P* value, Chi-Square *P* value or Fisher’s Exact Test *P* value as appropriate

**Table 2:** Results for linear mixed-effects and linear models of pain variables versus interaction of technique and time period, adjusting for repeated measurements over time

*Outcome*	*Interaction/Predictor*	*Period - hours*	***ESPB****N = 33 mean (SD)*	***WI****N = 34 mean (SD)*	*Mean difference*^a^*(95% CI)*	*Comparison P value*	*Interaction/Global P value*
Intraoperative fentanyl use	Technique		469.7 (198.4)	491.3 (265.4)	−21.6 (− 136.2, 93.0)		0.708
Rest pain	Period*Technique	0	1.6 (2.5)	1.9 (3.1)	−0.3 (−1.5, 0.9)	0.606	0.892
		1	3.3 (2.2)	3.8 (2.4)	−0.5 (− 1.7, 0.7)	0.382	
		24	2.4 (2.0)	3.0 (2.2)	−0.6 (−1.8, 0.5)	0.285	
Cough pain	Period*Technique	0	2.3 (3.3)	2.9 (3.5)	−0.6 (−2.0, 0.8)	0.375	0.953
		1	4.4 (2.4)	4.8 (2.8)	−0.4 (−1.7, 1.0)	0.595	
		24	5.3 (2.3)	5.9 (2.5)	−0.5 (−1.9, 0.8)	0.431	
Cumulative Fentanyl use	Period*Technique	1	117.6 (107.8)	104.1 (111.4)	13.5 (−200.0, 226.8)	0.900	0.911
		24	760.3 (682.1)	730.7 (527.3)	29.6 (− 183.8, 242.9)	0.783	
Total fentanyl used^b^	Technique		877.9 (731.9)	834.9 (557.0)	43.0 (− 273.7, 359.8)		0.787

*ESPB* denotes Erector Spinae Plane block, *WI* denotes wound infiltration, *PACU* Post anaesthetic care unit, *CI* Confidence interval

^a^The comparison is ESPB vs WI

^b^Total fentanyl used is the amount used during PACU and day one

**Table 3:** Complications

	**ESPB**	**WI**	**Fisher’s Exact Test* p*-value**
	***N* = 33**	***N* = 34**	
Ileus	3 (33%)	6 (67%)	0.48
Aspiration Pneumonia	1 (50%)	1 (50%)	1.00
Hypotension	1 (100%)	0 (0%)	0.49
Atelectasis	1 (50%)	1 (50%)	1.00

Data are presented as n (%)

*ESPB* denotes Erector Spinae Plane block, *WI* denotes wound infiltration

## Discussion

The main outcome of the study was that we found no treatment- related differences in NRS pain scores at rest and coughing in PACU or day one between the groups. There was no statistically significant difference found in mean total fentanyl use between ESPB and WI groups. There were no differences in adverse events or length of stay between the groups. Though we hypothesised that ultrasound-guided ESP block is superior to wound infiltration in providing superior pain relief, this was not confirmed by our findings.

Technically, we did not have any failures but had slight difficulty in three obese participants in the ESPB group requiring 120 mm needles to reach the plane. Complications related to LAST were not observed. Only one patient in the ESPB group had bradycardia at 24 h on the ward, but remained haemodynamically stable with unremarkable ECG. Tulgar et al. found 3 mild cases of LAST in ESPB patients [12]. However, as stated, the patients in our study did not show any such symptoms. There were two patients in WI group who developed bradycardia, one in the PACU and the other outside the 24 h study period, both with unremarkable ECGs.

We performed ESPB at T8 level, however we did not observe any clinical effects on dermatome sensory distribution on the anterior aspect of the chest. We used 20 ml of 0.5% ropivacaine each side and it is possible that this may be an inadequate volume leading to poor sensory block. The optimal volume may range from 20 to 30 ml [13]. Tulgar et al. in their case series performed ESPB at T8 level for laparoscopic surgeries and reported its analgesic benefits but failed to report sensory block [8]. Similarly Chin et al. performed ESPB at T7 level in four patients undergoing laparoscopic ventral hernia repair and reported reduced pain scores in the first 24 h and oral morphine consumption [5]. They reported dermatome spread from T6 to T12 in one of their patients. In our study, though patients achieved analgesia, we did not observe any dermatomal sensory block. We are unable to explain this finding. Peng et al., described that the ESPB has characteristics of differential blockade [14]. Analgesia without motor block along discernible cutaneous sensory block has been described [14]. A review on dermatomal analysis of case reports revealed variable results of ESPB dermatomal spread [15]. Due to its unpredictable dermatomal spread more clinical trials are required to assess this. A recent narrative review reports that the mechanism of ESPB is from the direct effect of LA via physical spread and diffusion to ESP and adjacent tissue compartments [16, 17]. It also highlights the unpredictability and variability that result from myriad factors [16, 17].

This limited LA spread may be due to the mechanical barrier of the intertransverse ligament, intertransversalis muscle, and/or superior costotransverse ligaments in the thoracic paravertebral space [18]. Only intertransverse and superior costotransverse ligaments are found in the thoracic region posing a possible obstacle [19]. Some authors reported benefits of technical refinements of ESPB such as double injection technique, multiple level injections and injecting near the costotransverse ligament in breast procedures, to improve LA diffusion into the paravertebral space [20–22]. There are no published trials on these new approaches for performing ESPB in abdominal surgery. Future clinical trials on this should be considered. A metanalysis on ESP found reduction in postoperative opioid consumption compared to control [23]. However, this study had significant heterogeneity.

There were a few limitations of our trial: It was conducted in a single centre, was single blinded and practice of WI may not be applicable in other settings. As there were no RCTs, sample size calculation was not possible prior to commencement of this study. We were optimistic in requiring a 2.5 points difference in pain scores between the groups. Nevertheless, given our findings, even using a minimally clinically important difference (MCID) of only 1 point as suggested by Myles et al. [24] may not have changed our overall outcomes. However, a substantially lower MCID would have made our study with current numbers underpowered. The volume we used (20 ml) may be low, higher volumes may produce more extensive physical spread.

In conclusion, this prospective, single-centre, randomised, open label study revealed that both WI and ESPB techniques were comparable in terms of pain scores and rescue opioid requirement during the first 24 h post-operatively. There were no differences in complications observed between the two techniques. As the ESPB appears to be more invasive, and requires expertise, local anaesthetic wound infiltration remains a more practical and relatively simple technique in laparoscopic colonic surgery.

## Data Availability

The datasets generated during and/or analysed during the current study are available from the corresponding author on reasonable request.

## Abbreviations

WI: Wound infiltration
ESPB: Erector Spinae Plane Block
PACU: Post anaesthetic care unit
LA: Local anaesthetic
LAST: Local anaesthetic systemic toxicity
NRS: Numerical Rating Score
PCA: Patient controlled analgesia
